# Visualization approaches to support healthy aging: A systematic review

**DOI:** 10.14236/jhi.v23i3.860

**Published:** 2016-10-10

**Authors:** Uba Backonja, Nai-Ching Chi, Yong Choi, Amanda K. Hall, Thai Le, Youjeong Kang, George Demiris

**Affiliations:** Department of Biomedical Informatics and Health Education, University of Washington School of Medicine, USA; Department of Biobehavioral Nursing and Health Systems, University of Washington School of Nursing, USA; Department of Biomedical Informatics and Health Education, University of Washington School of Medicine, USA; Department of Biomedical Informatics and Health Education, University of Washington School of Medicine, USA; Department of Biomedical Informatics and Health Education, University of Washington School of Medicine, USA; Department of Biobehavioral Nursing and Health Systems, University of Washington School of Nursing, USA; Department of Biomedical Informatics and Health Education, University of Washington School of Medicine, and Department of Biobehavioral Nursing and Health Systems, University of Washington School of Nursing, USA

**Keywords:** Aged, consumer health information, data display, informatics, visualization

## Abstract

**Background:**

Informatics tools have the potential to support the growing number of older adults who are aging in place. Many tools include visualizations (data visualizations and visualizations of physical representations). However, the role of visualizations in supporting aging in place remains largely unexplored.

**Objective:**

To synthesize and identify gaps in the literature evaluating visualizations (data visualizations and visualizations of physical representations) for informatics tools to support healthy aging.

**Methods:**

We conducted a search in CINAHL, Embase, Engineering Village, PsycINFO, PubMed, and Web of Science using *a priori* defined terms for publications in English describing community-based studies evaluating visualizations used by adults aged ≥ 65 years.

**Results:**

Six out of the identified 251 publications were eligible. Most studies described in the publications were user studies and all varied methodological quality. Three publications described visualizations of virtual representations supported performing at-home exercises. Participants found visual representations either (1) helpful, motivational, and supported their understanding of their health behaviours or (2) not an improvement over alternatives. Three publications described data visualizations that aimed to support understanding of one’s health. Participants were able to interpret data visualizations that used precise data and encodings that were more concrete better than those that did not provide precision or were abstract. Participants found data visualizations helpful in understanding their overall health and granular data.

**Conclusions:**

Few studies were identified that used and evaluated visualizations for older adults to promote engagement in exercises or understanding of their health. While visualizations demonstrated some promise to support older adult users in these activities, the studies had various methodological limitations. More research is needed, including research that overcomes methodological limitations of studies we identified, to develop visualizations that older adults could use with ease and accuracy to support their health behaviours and decision making.

## INTRODUCTION

By 2050, the older adult population (age ≥ 65 years) is estimated to double in the US and triple worldwide.^1,2^ Many older adults will likely live at home – in 2013, 26.8 million US house-holds were headed by older adults^3^ and approximately 80% of US older adults receiving long-term care services resided at home.^4^ Informatics tools can address the needs of older adults aging in place,^5^ including telehealth^6–8^ and smart home systems.^9,10^ Research has focused on the technical feasibility of these systems rather than on the effectiveness of visualizations that such systems generate. Development of tools with visualizations, including visualizations of data and virtual representations (e.g. environments and people)and tools’ roles in supporting healthy aging in place remain largely unexplored.

Data visualization is the visual representations of data, encoded using position, length, size and/or colour, among others, to reduce complexity and effectively communicate information to support discovery and understanding of patterns within data, decision making and memory.^11–14^ In health informatics, data visualizations can display longitudinal health information (e.g. historical vital sign or symptom data) and support health-related decision making and behaviours (e.g. using icons to convey disease risk, medication side effects or treatment benefits).^15–23^ Data visualization has been used to support clinical care^24,25^ and personal health tracking (e.g. quantifiedself.com/visualization).

Visualizations of physical representations include virtual environments (e.g. landscapes) and people, among others. With advancements in graphics and movement capture technologies used in gaming consoles (e.g. Xbox Kinect), interaction with physical representation visualizations is increasingly prevalent. Technologies providing these visualizations using movement capture can support older adults’ health and wellness.^26–29^

Unfortunately, few informatics tools with data or physical representation visualizations have been specifically developed to support older adults and the benefits of these visualizations have not been established. Also, it is unknown how data visualizations and visualizations of physical representations can be used to support community-dwelling older adults’ ability to understand and use information. The purpose of this systematic review was to synthesize and identify gaps in the literature regarding the evaluation of data visualizations and visualizations of physical representations included in informatics tools to support healthy aging in place.

## METHODS

Publications were eligible if they were published before 9 June 2015 and were full-text peer reviewed articles, described a study, took place in a community-based setting, included older adults aged ≥ 65 years, visualization users were older adults, included evaluation of visualizations, and were in English. We used the Preferred Reporting Items for Systematic Reviews and Meta-Analyses (PRISMA) statement to guide our reporting.^30^

Using a predetermined list of terms developed with a health sciences librarian (Supplemental Table 1), two researchers (YC, NCC) conducted searches independently in CINAHL, Embase, Engineering Village, PsycINFO, PubMed, and Web of Science. The two researchers met to compare results, which were identical. Compiled citations were uploaded into covidence.org, which pairs of researchers used to review each abstract (UB and NCC; YC and JK) and full-text article (UB and GD; AKH and NCC) for eligibility. The following information was abstracted from eligible publications: design, sample, description of comparison group, criteria for evaluating visualizations, and methods that researchers used to improve internal validity in their study designs and study results. Researchers noted limitations that publication authors identified and limitations not discussed by the authors.

## RESULTS

**Figure 1:**
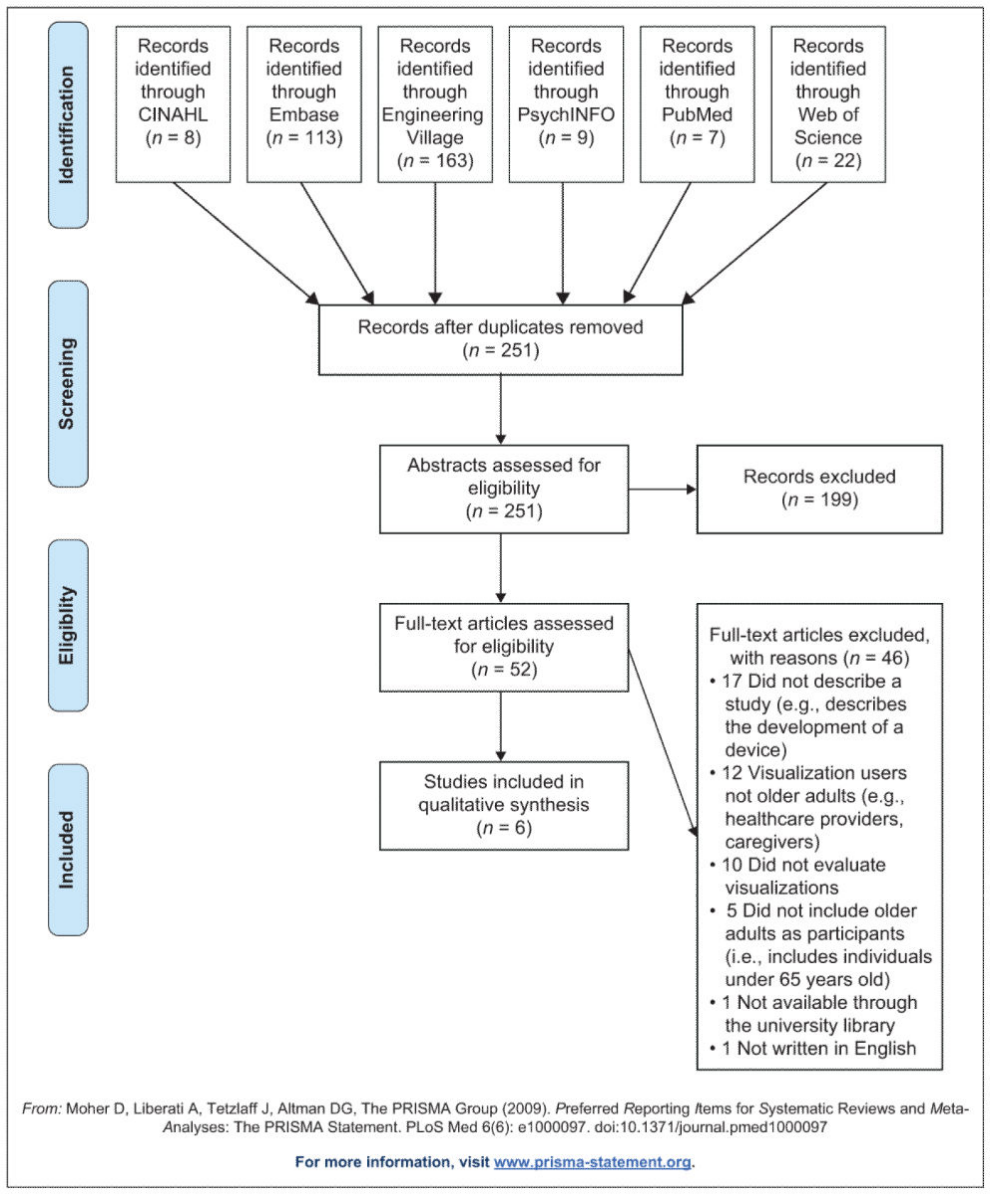
PRISMA flow diagram of the manuscript selection process

We identified 251 publications (Figure 1). Of those, 199 (79.3%) publications did not meet inclusion criteria and 52 (20.7%) were included for full-text review. Of the 52 full texts, 46 (88.5%) were excluded (e.g. older adults were not the visualization user). Six of the 52 (11.5%) met our inclusion criteria.^31–36^

### Study characteristics

Table 1 provides characteristics of the studies described in the six publications. Studies were observational user studies of visualization tools,^32,33,35^ quasi-experimental within-subject studies comparing the completion of exercises using a printed informational booklet or visualization^31,36^ or a heuristic evaluation of visualizations.^34^ Sample sizes ranged from two to 165. Among publications with demographic information, samples generally included older adults aged ≥ 65 years and participants were healthy or experiencing health problems (e.g. had a chronic disease). Studies were completed in Denmark, the United Kingdom, or the US and published in 2013–2015.

### Visualizations, evaluations and findings

Table 2 provides information about visualizations, their evaluations, and study findings. Visualizations either supported performing exercises via virtual using three-dimensional representations (e.g. virtual outdoor environments)^31,32,36^ or understanding of one’s health via data visualizations (e.g. graphs, charts or icons to represent quantitative data).^33–35^

#### Virtual representation visualizations to support exercises

Two virtual representation visualizations were developed that included mannequins and natural landscapes presented on screens with which participants interacted. Ayoade et al.^31^ and Uzor and Baillie^36^ developed animated visualizations to engage older adults in home exercises using human-like representations (mannequins). Participants wore sensors that collected information to provide visual feedback about their movements and proper posture using a real-time feedback mannequin and a guide mannequin, respectively. Weekly progress charts were provided to participants but not evaluated in the study. Mannequin visualizations were evaluated by comparing within-subject completion of exercises using an informational booklet followed by the mannequins. Ayoade et al.^31^ collected feedback via observation, semi-structured interviews and short questionnaires. Uzor and Baillie^36^ used a questionnaire and assessed differences in time to complete exercises when using the booklet and then the visualizations. Both studies demonstrated that the visualizations improved participants’ perceived confidence in performing exercises and ability to perform more controlled movements compared to when using the booklet. Participants found mannequins helpful to identify movement or position problems while completing exercises and motivated them to complete otherwise unexciting exercises. When timed, participants using the visualizations took longer to complete exercise repetitions compared to using the booklet.

Bruun-Pedersen et al.^32^ described a virtual outdoor environment projected on a monitor to support exercise engagement. Older adults rode exercise bicycles and viewed a virtual environment mimicking natural landscapes that changed while pedalling. No feedback about performance was given to participants. Researchers used openended interviews to assess participants’ experiences using the virtual environment. Most participants felt the environment enhanced their exercise experience and gave them energy and a sense of accomplishment. They felt the virtual environment could motivate them to exercise regularly or for a longer duration. Two of the ten participants with pain did not feel the virtual environment impacted their exercise engagement. Five of the ten participants stated the virtual environment did not match their interests or could become less engaging if novelty was lost.

#### Data visualizations to support understanding of one’s health

Three publications described studies in which researchers evaluated visualizations of quantitative health information in the form of graphs and icons. Gronvall and Verdezoto^33^ developed data visualizations to support participants’ understanding of blood pressure (BP) measurements. They created (1) three data visualizations (icon-based, bar charts, line charts) to provide a one-week BP overview and (2) four data visualizations (icon based, text based, speedometer and slider) to show daily BP measurements. Data visualizations were evaluated by (1) older adults who participated in a workshop in which they measured their BP for one week and interpreted visualizations of their BP data and (2) adults who completed an online survey. It is unclear how researchers presented the visualizations to the workshop participants; participants in the survey study viewed the visualizations within the web-based survey. Participants felt the data visualizations enhanced their understanding of BP measurements; however, they were concerned with visualization precision. For the one-week overview, participants positively responded to the line chart. For the daily view, participants noted that icons were simple yet lacked precision; they used text representations for precise values. Participants had mixed reactions towards the speedometer visualization, noting that there might be problems with the precision of interpreting the visualization.

Le et al.^34^ created a streamgraph (variant of stacked line-graph) and a radial plot (a circle that represents a 24-hour clock) using motion data from sensors worn for six months by older adults in their apartments. Visualizations were developed based on interview data with older adults who wore the sensors, cognitive perceptual visualization guidelines, the emotional design principles of Norman,^37^ and Shah and Hoeffner’s model of information visualization processing.^38^ For evaluation, researchers recruited gerontology experts to review the data visualizations presented digitally on a laptop and provide heuristic-based feedback. Participants mostly understood the spatial and temporal component of the stream graph and radial plot visualizations. They found the radial plot easier to understand than the streamgraph to compare components within the visualization and understand granular data.

Le et al.^35^ developed three interactive data visualizations to provide information about older adults’ overall wellness and social, physical, cognitive, and spiritual health. The data visualizations included a bar graph, a radial plot (area represented score; different from the radial plot described in the previous paragraph) and a light ball metaphor (a circle for which the size and brightness encoded data). Researchers designed the data visualizations based on findings from previous research, focus groups with gerontology experts and heuristic design guidelines. To evaluate the visualizations, they held a focus group with older adults who used then reported on their experiences with the visualizations, which were presented on paper. Participants used the data visualizations first for a holistic perspective and then looked at details. They felt there was too much information displayed in the visualization and were confused by data abstractions (e.g. light ball metaphor). It was difficult for participants to notice differences in sizes and brightness encodings. Participants appreciated separation of visualizations for different components of wellness. They felt there was potential for data visualizations to support assessments of their wellness and promote shared decision making with healthcare providers.

### Methodological quality

#### Study design

Four publications described studies that assessed participants’ opinions about visualizations. These studies provide information about potential value of visualizations but do not compare visualizations to alternatives. Two publications described studies that used within-subject designs to compare the current standard of providing exercise information (a booklet) to their visualization tool, providing data comparing opinions and abilities after using the booklet and visualization tools. All participants first used the booklet and then the visualization tool; therefore, participants were aware of and had performed the exercises by the time they started exercises with the visualization tool. This ordering effect could have impacted participants’ opinions about and ability to perform subsequent exercises.

#### Sample

Most studies had sample sizes ≤ 10. While researchers can detect usability issues using five to eight participants^39^, conclusions drawn from experimental studies with small sample sizes should be made with caution; it is possible that samples were not big enough to detect differences in performance interpreting data). Most studies had incomplete information about participants’ gender, socioeconomic status, education, health status, and technology use, limiting assessments of the generalizability of findings.

#### Visualization development

Researchers varied in amounts of evidence they used to guide development of their visualizations. They varied from using one previously published paper to using a combination of sources (e.g. previous research, visualization guidelines and a theoretical model). It is possible that the number and types of evidence researchers used to develop the visualization could have impacted their efficacy.

#### Visualization evaluation

Most studies included interviews with or gathered feedback from users. Fewer studies included questionnaires; information was not provided in the publications about questionnaire reliability and validity, whether researchers developed the questionnaires, or if questionnaire development was guided by a theory or framework. Interview and questionnaire methods are adequate for providing qualitative and/or quantitative feedback; however, most studies using these methods did not describe providing a usual information or data presentation option (i.e. a control comparison) for participants to which to compare. Participants provided feedback on one or multiple visualizations designed by researchers. These publications do not provide insights on if and how the visualizations compare to usual data presentations. Two publications^31, 36^ described studies in which researchers compared exercise completion using a traditional method (information booklet; a control comparison) versus visualizations (real-time feedback and guide mannequins). However, these studies were of within-subject design and did not change the order in which participants received the booklet or visualization tool. It is difficult to determine why there were differences in time to complete the exercise repetition. Finally, all studies appeared to be short in duration making it difficult to determine if (1) learning curves for the health-related visualizations were overcome with prolonged use or (2) older adults engaged in sustained use of certain exercise and health-related visualizations.

## DISCUSSION

We summarized the current published research evaluating visualizations of physical representations to support exercise engagement and data visualizations for understanding one’s health incorporated into tools for older adults in the community. Studies evaluating virtual environments or human representations (three publications^31,32,36^) showed potential to promote exercise engagement. Older adults found them motivating, which may be important among older adults who find it difficult to engage in activities due to impaired physical abilities. These studies were limited methodologically in several ways, including study duration, making it is difficult to draw clear conclusions about the efficacy of the visual representations.

Studies of data visualizations to better understand one’s health (three publications^33–35^) also showed promise, although they had several methodological limitations that should be taken into consideration when interpreting the findings. Among standard data visualizations, line and bar graphs were developed by study researchers to show quantitative health data. Previous quantitative data visualization research indicates that position and length – how line and bar graphs are represented, respectively—support more accurate data interpretation.^40,41^ Researchers of the studies we identified in this review (e.g. Gronvall and Verdezoto^33^) found that line and bar graphs (optimal encodings) were more understandable among their participants than alternatives such as abstract icons. Previous data visualization research also indicates that area and hue are harder to interpret than position and length. In research to understand graphical perception for older adults, Le et al.^42^ found that participants were not as quick or accurate in understanding stacked bar charts and pie graphs (encode area; less optimal) compared to bar charts (encodes length; optimal). During their studies, Le et al.^35^ found that older adults who viewed the light balls metaphor visualization (area and hue encodings; less optimal) had difficulty identifying differences between balls. It is possible to use area to represent something familiar. For example, Le et al.^34^ used circle radial plots representing a 24-hour clock to encode temporal data, which older adults preferred to the streamgraph. Later, Le et al.^35^ used radial plots more similar to pie charts in which areas and arc lengths are compared, which older adults found confusing. Thus, it is possible for researchers to investigate (1) the validity of previous data visualization research in the context of consumer health informatics tools for older adults and (2) new approaches to visualize quantitative data in ways that optimize older adults’ familiarity with certain objects.

The speedometer is another representation using arc length to encode quantitative data in a familiar way. However, this visualization could be difficult to interpret – speedometers visualize speed, which may not map to health and wellness characteristics. Gronvall and Verdezoto^33^ in their speedometer visualization provided (1) general BP categories (e.g. low and normal) across the speedometer arc encoded with colour and (2) BP values in a box that had colours identically to the category on which the needle was positioned. Although redundant encodings were included, participants felt the speedometer lacked precision, possibly because arc length is not as optimal in encoding quantitative data as position or linear length.^40,41^ While study authors did not provide information about whether participants preferred speedometers to a slider (similar to a stacked bar graph, a more optimal encoding than arc length), participants stated they found the slider useful.

Participants in the three health data visualizations appeared to have had different encoding preferences depending on data granularity. They preferred overviews, were overwhelmed if too much data were presented, and wanted ways to access precise data.^33,35^ One solution is to provide static views of overviews and granular data, as in Gronvall and Verdezoto’s work,^33^ or interactive visualizations to allow viewing an overview, zooming and filtering of data and accessing to detailed information on demand,^43^ as in Le et al.’s work.^35^

Future research could build on the current literature by addressing methodological limitations of studies included in this review. This includes using multiple sources of evidence to inform the design of visualizations to guide researchers towards more understandable visual encodings; using designs that allow comparison between usual standards and visualizations; including larger, diverse samples; allowing for extended use of visualizations; and including validated measures and interviews to evaluate visualizations. Within-subject studies could randomly assign the order in which participants used current standards and novel visualizations. Also, researchers should be cognizant of how evaluate visualizations for older adults. Le et al.^44^ evaluated three approaches to assess interactive visualizations for older adults. They found the evaluation methods varied in differences with task completion time and accuracy. In addition, researchers could consider assessing graph literacy and numeracy in addition to comprehension when evaluating visualizations. Nayak et al.^45^ found that older adult prostate cancer patients who were highly educated and had high health literacy varied in their comprehension of a dashboard that included a table, line graph and bar graph depending on their graph literacy and numeracy. Researchers could further investigate evaluation techniques and consider using evaluation methodology when assessing their visualization tools.

## LIMITATIONS

We identified that few publications and studies were heterogeneous in design. Therefore, we were unable to aggregate data across studies. We consulted with a health informatics librarian to develop the search strategy; however, we may not have identified all relevant articles.

## CONClUSION

We identified six studies in which researchers evaluated visualizations of physical representations to promote engagement in exercises or data visualizations for understandings of one’s health. Visualizations show promise in supporting the health and wellness of community-dwelling older adults; however, because of the low number of publications we identified and the methodological limitations of studies described in these publications, caution should be made in interpreting and extending findings from these studies. Future research could build on this currently literature to develop informatics tools including visualizations that older adults could use with ease and accuracy. With the projected rise of older adults living at home in the coming decades, more home-based tools using data visualizations and visualizations of physical representations are needed. Informatics tools may provide that support; however, developers of informatics tools for older adults’ in the community could benefit from developing evidence-based visualizations that they then evaluate.

## Supplementary Material

01

## Figures and Tables

**Table 1:** Study characteristics of studies to evaluate visualizations used in consumer health technologies to support older adults living in the community.

Citation	Country	Sample	Sample description
Ayoade et al.^31^	United Kingdom	Study 1: *n* = 3 Study 2: *n* = 3 Study 3: *n* = 2 Study 4: *n* = 3	≥ 60 years old Mean age (years): Study 1 = 68, Study 2 = 71, Study 3 = 79, Study 4 = 63 Genders: Study 1 = two males/one female, Study 2 = three males, Study 3 = one male/one female, Study 4 = 2 males/1 female Either had knee replacement surgery in the past 18 months or experienced ≥ one fall within the past year

Bruun-Pedersen et al.^32^	Denmark	*n* = 10	66–97 years old two males/eight females Seeing a physical therapist Experience with and ability to ride a manuped

Gronvall and Verdezoto (2013)	Denmark	Phase 1: *n* = 10^2^ Phase 2: *n* = 10 Phase 3: *n* = 165	Phase 1: Mean age 61.8 years; healthy *n* = 1, taking medication preventively *n* = 3, chronic disease *n* = 3, recently had arterial thrombosis or cancer and participating in physical therapy = 3 Phase 2: 65–84 years old; self-perceived as being ‘healthy’ Phase 3: 22–83 years old; own a health-monitoring device *n* = 86

Le et al.^34^	United States	Phase 1: *n* = 8^3^ Phase 2: unknown	Phase 1: ≥ 65 years old; spoke English Phase 2: Gerontology experts

Le et al.^35^	United States	*n* = 30	≥ 62 years old; 8 males/22 females; spoke English Resided in private apartments or assisted living facilities

Uzor and Baillie^36^	United Kingdom	Study A: *n* = 4^4^ Study B: *n* = 3 Study C: *n* = 2 Study D: *n* = 2	At least a high school education Had previous experience with home exercises Mean age (years): Study A = 71, Study B = 68, Study C = 78, Study D = 79 Genders: Study A = two males/two females, Study B = two males/one female, Study C = two females, Study D = one male/one female

1 Studies 1 and 2 were completed in a laboratory among participants who experienced a fall or had knee replacement surgery, respectively. Studies 3 and 4 were completed in participants’ homes among those who experienced a fall or had knee replacement surgery, respectively

2 Phase 1 was to understand how older adults maintain awareness of health status. In phase 2, older adults measured their BP and provided feedback on visualizations of their BP data. Phase 3 consisted of a web survey of adults assessing self-monitoring needs.

3 Phase 1 was a pilot study in which community-dwelling older adults used a sensor system in their apartments for six months. Phase 2 consisted of interviews with gerontology experts to gain heuristic-based feedback on visualizations developed by the researcher using Phase 1 data.

4 Study A was conducted in a laboratory and assessed exercise-based games. Study B was conducted in a laboratory and assessed visualizations of user movements. Study C assessed games in participants’ homes. Study D assessed visualizations in participants’ homes.

**Table 2:** Visualization intervention, evaluation, and results of observational studies to evaluate visualizations used in consumer health technologies to support older adults living in the community.

Citation	Visualization(s)	Intervention	Visualization evaluation method	Results
Ayoadeetal.^31^	Different visualizations for knee replacement surgery and fall participants. Visual feedback using guide and real-time feedback mannequins to show users how and where to place body sensors, exercises to be completed, and feedback about exercise performance including a weekly progress report. Consulted with falls experts prior to developing the visualization	In either a lab or at home, participants reviewed an informational booklet and used a visualization tool while performing rehabilitation exercises	Observations Semi-structured interviews Short questionnaires	The visualization tool improved confidence in executing the exercise program. The visualization tool encouraged slower, more controlled movements compared to the booklet use. Participants appreciated the weekly chart feature as a tool that allowed them to assess their performance over time.

Bruun-Pedersen et al.^32^	A virtual environment application describing landscapes that changed as participants used the exercise bicycle to give the impression that participants were cycling through the landscapes. Developed visualization based on previous literature regarding interactions with virtual environment-related technologies.	Participants used the exercise bike and if they wanted to, focus on the screen that provided the Virtual Environment feature.	Open-ended interviews	Seven participants preferred the virtual environment; three participants did not prefer it. Participants overall were enthusiastic about the Virtual Environment feature, that it enhanced the exercise routine, and motivated to exercise regularly or for a longer duration. It provided (1) a feeling of being outside, (2) a sense of accomplishment and (3) them with energy. Two participants with pain did not feel that the virtual environment made a positive difference. Five participants stated that improvements could be made (the environment did not match their interests or was redundant; novelty of the environment could be lost).

Gronvall and Verdezoto^33^	Three different visualizations used to show weekly BP overviews (icon based, bar charts, line charts). Four visualizations used to show daily BP (icon based, text based, speedometer, slider). Designs guided by Beaudin and colleagues (2006).*	Participants performed BP self- measurement for 1 week and interpreted BP visualizations (phase 2)	Workshop feedback (phase 2) Web-based survey of adults (phase 3)	Visualizations helped enhance understanding of BP measurements. For the weekly view, the line chart was preferred. For the daily view, participants found icons simple although it lacked in precision; they used text representations for precise values. Mixed response towards the speedometer visualization; participants noted that precision might be an issue. Overall, participants were concerned with precision of measurements in the visualizations.

Leetal^34^	Two visualizations of passive sensor data regarding participants' motion within their apartments: a streamgraph (variant of stacked bar graph) displayed longitudinal total sensor activity distributed by location within the home, thickness of each layer corresponds amount of sensor activity, and a radial plot, a clock-like display of a 24-hour period of sensor data. Researchers developed visualizations using participant interview data, cognitive perceptual visualization guidelines, the emotional design principles of Norman^37^ and Shah and Hoeffner's model of information visualization processing.^38^	Community-dwelling older adults used a passive sensor system in their apartments for six months (phase 1)	Interviews with gerontology experts for heuristic-based feedback (phase 2)	Overall, participants understood the spatial and temporal component of the visualizations. The radial plot was easier to understand than the streamgraph for comparing components in the visualization and understanding granular data.

Leetal.^35^	Researchers developed three interactive visualizations - a bar graph diagram, a radial plot, and a light ball metaphor - that provided information about overall wellness and social, physical, cognitive, and spiritual health. Visualizations were guided by previous research and suggestions from gerontology researchers.	Focus groups with older adults in which they interacted with the visualizations	Interview questions during focus groups	Participants noted potential for visualizations to support assessments of their wellness and promote of shared decision making with healthcare provider. They wanted to identify interventions they could use to address trends in longitudinal data. Participants used visualizations first for a holistic perspective then looked at details. Participants thought there was too much information displayed in the visualization and were confused by data abstractions (e.g. radial plot, light ball metaphor). Participants found it difficult to notice differences in sizes and brightness. They appreciated that separation of visualizations based on different components of wellness.

Uzor and Baillie^36^	Researchers developed two animated visualizations of a mannequin: a guide mannequin that demonstrated movements for each exercise (passive feedback); a guide mannequin and a mannequin that showed users' movements (real-time feedback). Researchers also developed games that incorporated participants' movements. Developed visualizations after consulting with older adults and experts in falls and physiotherapy.	In each study, participants completed exercises using an instructional booklet then repeated exercises while wearing body-worn sensors and using either the games or visualization tool	Compared time taken to complete one exercise repetition using the booklet vs. the visualization tool Questionnaire	Participants using the visualization tool on average took longer to complete each exercise repetition compared to those using the booklet (6.58 versus. 5.66 seconds). They found the guide mannequin useful in identifying problems while completing exercises. Participants agreed that seeing exercise visualizations improved their understanding about rehabilitation and felt that visualizations made it hard for them to ignore completing exercises perceived of as unexciting.

* Beaudin JS, IntilleSS, Morris ME. To track or not to track: user reactions to concepts in longitudinal health monitoring. JMIR. 2006;8(4):e29
