# Supplementary material for: Visualization approaches to support healthy aging: A systematic review
Source: J Innov Health Inform. Author manuscript; Available in PMC 2017 Jan 9. (PMC5222528; doi:10.14236/jhi.v23i3.860)
Supplement: 01 [file NIHMS818342-supplement-01.pdf]

## SUPPLEMENTAL TABLE 1

**Predetermined list of search terms used for this systematic review to identify publications in which researchers evaluated visualizations for health technologies to support the health and wellness of elders living in the community**

| Database | Search terms |
|---|---|
| CINAHL | (visualization* OR visualisation) AND (informatics OR computer OR device OR technology OR application* OR instrument* OR sensor* OR monitor* OR track* OR interface OR graphic* OR presentation OR analytics OR display OR user-computer OR human-computer OR computer interaction OR human centered) AND (elder* OR older adult* OR geriatric* OR senior*) AND (consumer OR home OR community) |
| Embase | (visualization* OR visualisation) AND ('computer'/exp OR 'devices'/exp OR 'technology'/exp OR 'information processing'/exp OR 'monitor'/exp OR presentation OR analytics OR 'imaging and display'/exp OR 'human centered') AND ('aged'/exp OR 'geriatrics'/exp) AND ('consumer'/exp OR 'home'/exp OR 'community'/exp) |
| Engineering Village | (visualization* OR visualisation) AND (informatics OR computer OR device OR technology OR application* OR instrument* OR sensor* OR monitor* OR track* OR interface OR graphic* OR presentation OR analytics OR display OR user-computer OR human-computer OR computer interaction OR human centered) AND (elder* OR older adult* OR geriatric* OR senior*) AND (consumer OR home OR community) |
| PsychInfo | (visualization* OR visualisation) AND (informatics OR computer OR device OR technology OR application* OR instrument* OR sensor* OR monitor* OR track* OR interface OR graphic* OR presentation OR analytics OR display OR user-computer OR human-computer OR computer interaction OR human centered) AND (elder* OR older adult* OR geriatric* OR senior*) AND (consumer OR home OR community) |
| PubMed | (visualization* OR visualisation) AND (informatics OR computer OR device OR technology OR application* OR instrument* OR sensor* OR monitor* OR track* OR interface OR graphic* OR presentation OR analytics OR display OR user-computer OR human-computer OR computer interaction OR human centered) AND (elder* OR older adult* OR Aged"[Mesh] OR "Aged, 80 and over"[Mesh] OR geriatric* OR senior*) AND (consumer OR home OR community) |
| Web of Science | (visualization* OR visualisation) AND (informatics OR computer OR device OR technology OR application* OR instrument* OR sensor* OR monitor* OR track* OR interface OR graphic* OR presentation OR analytics OR display OR user-computer OR human-computer OR computer interaction OR human centered) AND (elder* OR older adult* OR geriatric* OR senior*) AND (consumer OR home OR community) |
